# Ultrafine metallic Fe nanoparticles: synthesis, structure and magnetism

**DOI:** 10.3762/bjnano.1.13

**Published:** 2010-12-03

**Authors:** Olivier Margeat, Marc Respaud, Catherine Amiens, Pierre Lecante, Bruno Chaudret

**Affiliations:** 1 Université de Toulouse, LCC - CNRS, 205, route de Narbonne, 31077 Toulouse Cedex 04 - France. Tel: +33 (0) 5 61 33 31 82; Fax: +33 (0) 5 61 55 30 03; 2 Université de la Méditéranée, Faculté des Sciences, GCOM2, 163 Avenue de Luminy, 13288 Marseille Cedex 09 - France. Tel: +33 (0) 6 17 24 81 15; 3 LPCNO, INSA, 135 avenue de Rangueil, 31077 Toulouse Cedex 04 - France. Tel: +33 (0) 5 61 55 96 48; Fax: +33 (0) 5 61 55 96 97; 4 CEMES - CNRS, 29 rue Jeanne Marvig, 31077 Toulouse Cedex 04 - France. Tel: +33 (0)5 62 25 78 51; Fax: +33 (0)5 62 25 79 99

**Keywords:** iron nanoparticles, magnetic properties, organometallic synthesis, size effects, structure

## Abstract

The results of the investigation of the structural and magnetic (static and dynamic) properties of an assembly of metallic Fe nanoparticles synthesized by an organometallic chemical method are described. These nanoparticles are embedded in a polymer, monodisperse, with a diameter below 2 nm, which corresponds to a number of around 200 atoms. The X-ray absorption near-edge structure and Mössbauer spectrum are characteristic of metallic Fe. The structural studies by wide angle X-ray scattering indicate an original polytetrahedral atomic arrangement similar to that of β-Mn, characterized by a short-range order. The average magnetic moment per Fe atom is raised to 2.59 µ_B_ (for comparison, bulk value of metallic Fe: 2.2 µ_B_). Even if the spontaneous magnetization decreases rapidly as compared to bulk materials, it remains enhanced even up to room temperature. The gyromagnetic ratio measured by ferromagnetic resonance is of the same order as that of bulk Fe, which allows us to conclude that the orbital and spin contributions increase at the same rate. A large magnetic anisotropy for metallic Fe has been measured up to (3.7 ± 1.0)·10^5^ J/m^3^. Precise analysis of the low temperature Mössbauer spectra, show a broad distribution of large hyperfine fields. The largest hyperfine fields display the largest isomer shifts. This indicates a progressive increase of the magnetic moment inside the particle from the core to the outer shell. The components corresponding to the large hyperfine fields with large isomer shifts are indeed characteristic of surface atoms.

## Introduction

Progress in both experimental techniques and theoretical calculations over the past ten years have allowed the development of precise studies on the influence of size reduction on the magnetic properties of nanoparticles (NPs) down to the nanometer scale. A first spectacular result was the observation of the enhancement of the atomic magnetic moment in NPs of classical 3d ferromagnetic metals [1–4]. More surprisingly, the study of small Rh NPs revealed a paramagnetic to ferromagnetic phase transition induced by size reduction for clusters containing less than 40 atoms [5]. Band structure calculations have investigated the role of size reduction and demonstrated that it promotes a narrowing of the magnetic bands and thus an increase of the spin polarisation, associated to an enhancement of the orbital contribution [6–9]. However, even if these tendencies are now well established, there is some disparity in the experimental results, even in the case of the ferromagnetic 3d metals. In the case of free-standing Fe clusters, Billas and coworkers have demonstrated the enhancement of the magnetic moment µ_Fe_ when the cluster contains less than 1000 atoms [2–3]. In this size range some oscillations of µ_Fe_ with cluster size have also been revealed. Similarly, supported α-Fe NPs with diameters down to 2 nm show an enhancement of the hyperfine field *B*_Hyp_, indicative of enhanced µ_Fe_ [10–12]. Recent careful measurements, by X-ray magnetic circular dichroism (XMCD) [13–16], consistently indicate an increase in the ratio of the orbital magnetic moment over the spin magnetic moment. However, different values have been reported, from µ_L_/µ_S_ = 0.1 for 2 nm size selected clusters deposited on Si substrates [15], 0.15 for size selected clusters containing less than 10 atoms deposited on a Ni surface [13] and up to µ_L_/µ_S_ = 0.3 for Fe islands on a Au surface [16]. For all these systems, the structure of the clusters and the influence of the substrate, which could both modify the electronic band structure, remain uncertain. This could explain the disparities observed in the experimental results. The theoretical investigations carried out so far were restricted to free clusters and therefore cannot explain all these experimental results. Calculations of the orbital contribution lead to an enhanced µ_L_/µ_S_ ratio compared to the bulk value, but this enhancement is smaller than those estimated from XMCD measurements [9]. Interestingly, calculations by Pastor et al. demonstrate that large spin moments can be found for Fe clusters, depending on their structural arrangement [6].

In summary, since the magnetic properties may be strongly influenced by both their crystal structure and interactions with the substrate, it is important to develop new synthetic approaches which could allow extensive magnetic and structural investigations. In this respect, a chemical approach could be productive enough to afford NPs for both characterization and further use. For the past ten years we have developed a new method for the synthesis of metal NPs based on an organometallic approach [17]. We have, for example, shown that cobalt NPs prepared by the decomposition of an organometallic precursor under mild conditions in the presence of a stabilising polymer exhibit physical properties similar to those of free cobalt clusters [18].

In this article, we report the chemical synthesis of well-isolated Fe NPs embedded in a polymer, with diameters of less than 2 nm. The structural and chemical properties have been investigated by transmission electron microscopy, wide angle X-ray scattering (WAXS), and X-ray absorption near-edge structure (XANES). Preliminary results of this work have already been published [19]. Here a more detailed study of the magnetic properties is presented including Mössbauer spectrometry, ferromagnetic resonance (FMR) and superconducting quantum interference device (SQUID) measurements (static and AC susceptibility).

## Results and Discussion

### Synthesis and structural studies

**Figure 1:**
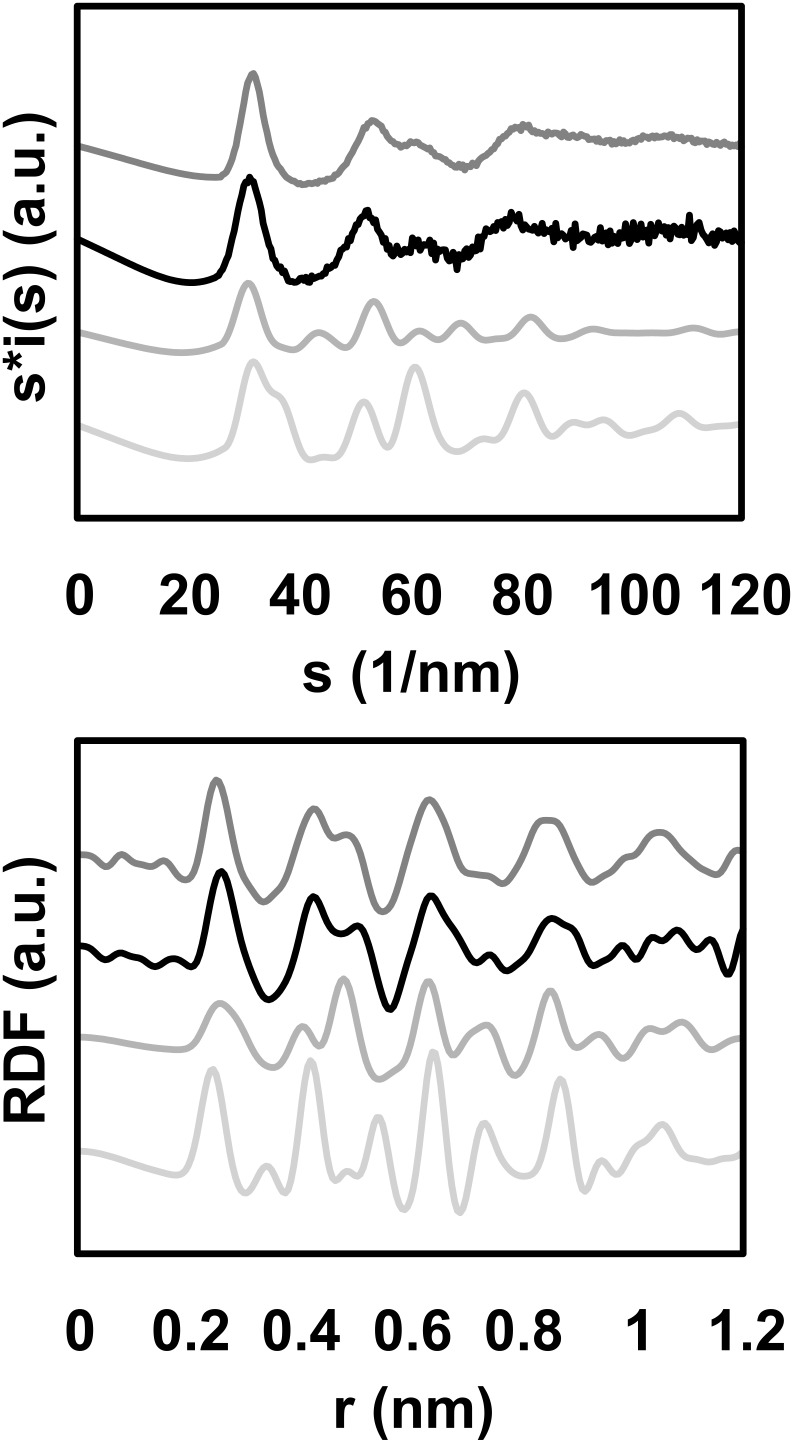
WAXS diagram (top) and the related RDF (bottom). Black: experimental spectra of Fe NPs taken at room temperature; dark grey (top): experimental spectra of Co NPs taken at room temperature; medium grey: α-Fe (bcc) model; light grey (bottom): γ-Fe (fcc) model.

The precursor chosen for the synthesis is {Fe[N(SiMe_3_)_2_]_2_} (**1**), which has previously been used for preparing self-organized iron nanocubes [20]. Furthermore, upon reduction with dihydrogen, it will generate, as the sole by-product an amine, a ligand previously shown to possess no influence on the magnetic properties of small metal particles [21]. The NPs were synthesized by dissolving the precursor **1** in a solution of polydimethylphenylene oxide (PPO) in toluene. After heating at 110 °C for 12 h under 3 bar H_2_, the reaction mixture turned black. The solvent and volatile byproducts were then removed at reduced pressure. The black residue consists of Fe NPs embedded in the polymer. This material, the Fe content of which can be determined by chemical analysis, may be used without further purification for physics measurements. All the samples were prepared in a glove box to prevent oxidation. The particles were characterized by transmission electron microscopy (TEM), wide angle X-ray scattering (WAXS) [22], and X-ray absorption near-edge structure (XANES). TEM micrographs show the presence of well-dispersed small particles of ca. 1.8 nm mean size with a narrow size distribution (15%). Interestingly, the WAXS diagram (Figure 1, top) and the radial distribution function (RDF, Figure 1, bottom) demonstrate that the particles do not adopt any of the bulk Fe structures (lower curves). The most peculiar points are as follows: in real space, a broad first peak, indicative of a large dispersion of metal–metal distances, and the absence of the peak at *d*√2 associated with octahedral sites in close-packed structures (with *d* the average metal–metal distance); in reciprocal space, the splitting of the second peak often observed in amorphous metals [23], including Fe [24–25], which also points to the absence of octahedral sites and thus suggests a polytetrahedral atomic arrangement with a very short periodicity.

This therefore excludes not only the close-packed fcc structure, but also many non-periodical structures commonly encountered in small particles, such as icosahedra or decahedra containing more than 50 atoms. It does not exclude the bcc structure, since the related RDF also exhibits a broad first peak and no peak at *d*√2. However, this function clearly does not match the experimental one over the complete range of distances (see Figure 1). In view of the WAXS diagram, the bcc structure can be also discounted since the experimental diagram does not present any intermediate peak for s in the range of 40–50 nm^−1^.

**Figure 2:**
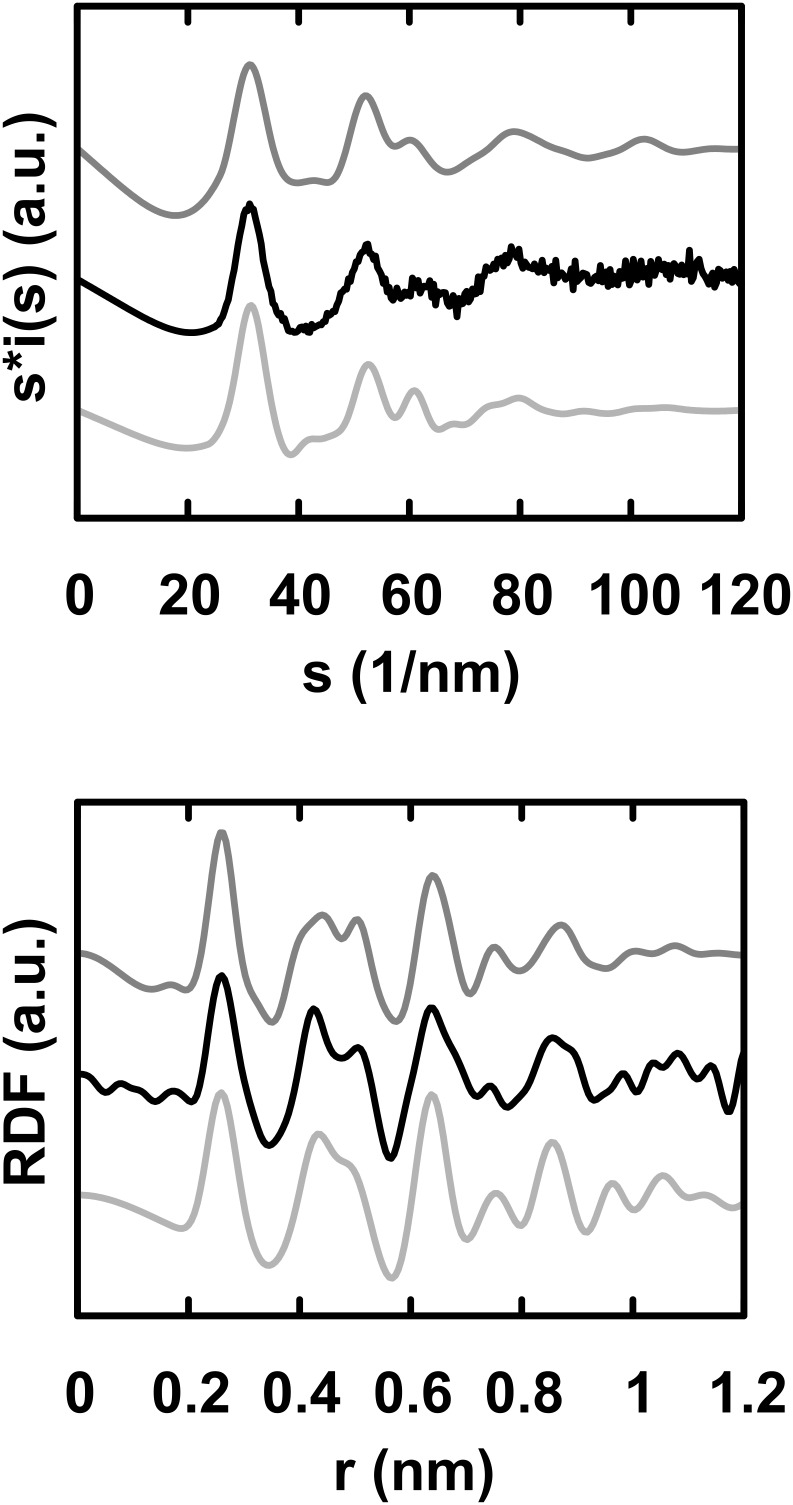
WAXS diagram (top) and the related RDF (bottom). Black: Fe NPs taken at room temperature; dark grey (top): Mn beta model; medium grey (bottom): van de Waal model.

Interestingly, both the WAXS diagram and the RDF resemble the corresponding curves obtained for Co NPs of 1.6 and 2.0 nm mean sizes prepared by a similar procedure and which were suggested to adopt a non-periodic polytetrahedral atomic arrangement. Such arrangements are locally ordered but lack the extended coherence length of regular structures. Different growth schemes lead to very close distance distributions, e.g., the shell-over-shell growth proposed for quasicrystalline alloys, or the disordered assembling of elementary icosahedra proposed for amorphous metals; both schemes adequately fit the experimental data [22]. As an illustration, Figure 2 displays the curves calculated from a van de Waal model (bottom curve). The first metal–metal distance ranges from 242.2 to 301.0 pm, leading to a structure locally more compact than in the bulk.

Interestingly, a small cluster based on the β-Mn structure leads also to a good agreement with the experimental data, both in the real (lower curve) and reciprocal spaces (upper curve) [26]. This structure, recently attributed to Co NPs (ε-Co) [27–28], displays several non-equivalent sites in distorted tetrahedral environments [26], which account for the large distribution of metal–metal distances and the splitting of the second peak observed in reciprocal space. The best fit between the calculated and experimental curves was obtained after contracting all distances occurring in the β-Mn structure by a factor of 1%, leading to metal–metal distances ranging from 236.4 to 267.9 pm, once again pointing to a locally more compact packing of iron atoms even if the overall calculated density for this model is 7.68 g/cm^3^, i.e., lower than that of bulk iron.

**Figure 3:**
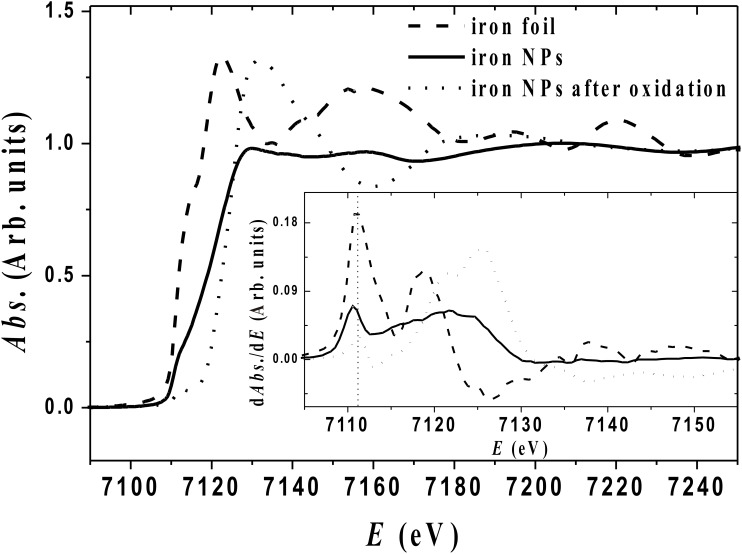
XANES spectra taken at room temperature for metallic Fe NPs, compared to a Fe foil reference, and intentionally oxidized Fe NPs. Inset displays the derivative of the absorption.

XANES and EXAFS are other powerful tools for the study of short range order [29]. We therefore carried out these measurements at the iron K-edge at room temperature. Figure 3 shows the data obtained for the iron NPs and an iron foil used as a reference, and the first derivative is shown in the inset of Figure 3. In both cases, the K-edge absorption, determined as the energy of the maximum of the first derivative, starts at 7111 eV and 7110.5 eV for α-Fe and NPs, respectively. These values are in agreement with those reported earlier [30]. In contrast, the third curve, which corresponds to the signal recorded after exposure of the NPs to air, displays a pre-edge characteristic of an iron oxide [30–32].

For the two metallic phases, the shapes of the edge itself are however, quite different. The second shoulder and the maximum of the absorption are shifted toward higher energies for NPs as compared to the reference. Unfortunately, the EXAFS signal is strongly damped, which prevents further analysis of the higher energy part. Notwithstanding, the results (both shape and damping) are consistent with published data on amorphous iron [31], thus exhibiting metallic NPs without long-range order. The structural determination is therefore not unequivocal. However, this study demonstrates NPs with a short-range order similar to β-Mn with a local polytetrahedral atomic arrangement with areas both more and much less dense than in bulk structures. It is noteworthy that the possible growth modes (atom per atom or cluster per cluster) are consistent with the synthetic procedure. Indeed, formation of a seed and its subsequent growth by random dense packing of atoms, generated during the hydrogenation of the iron precursor, can easily coexist in solution with a growth process involving the coalescence of small clusters. This emphasises the importance of the solution phase synthesis for the trapping of unstable intermediates and the growth of metastable structures often kinetically favoured.

### Magnetic properties

#### A. Mössbauer spectra

**Figure 4:**
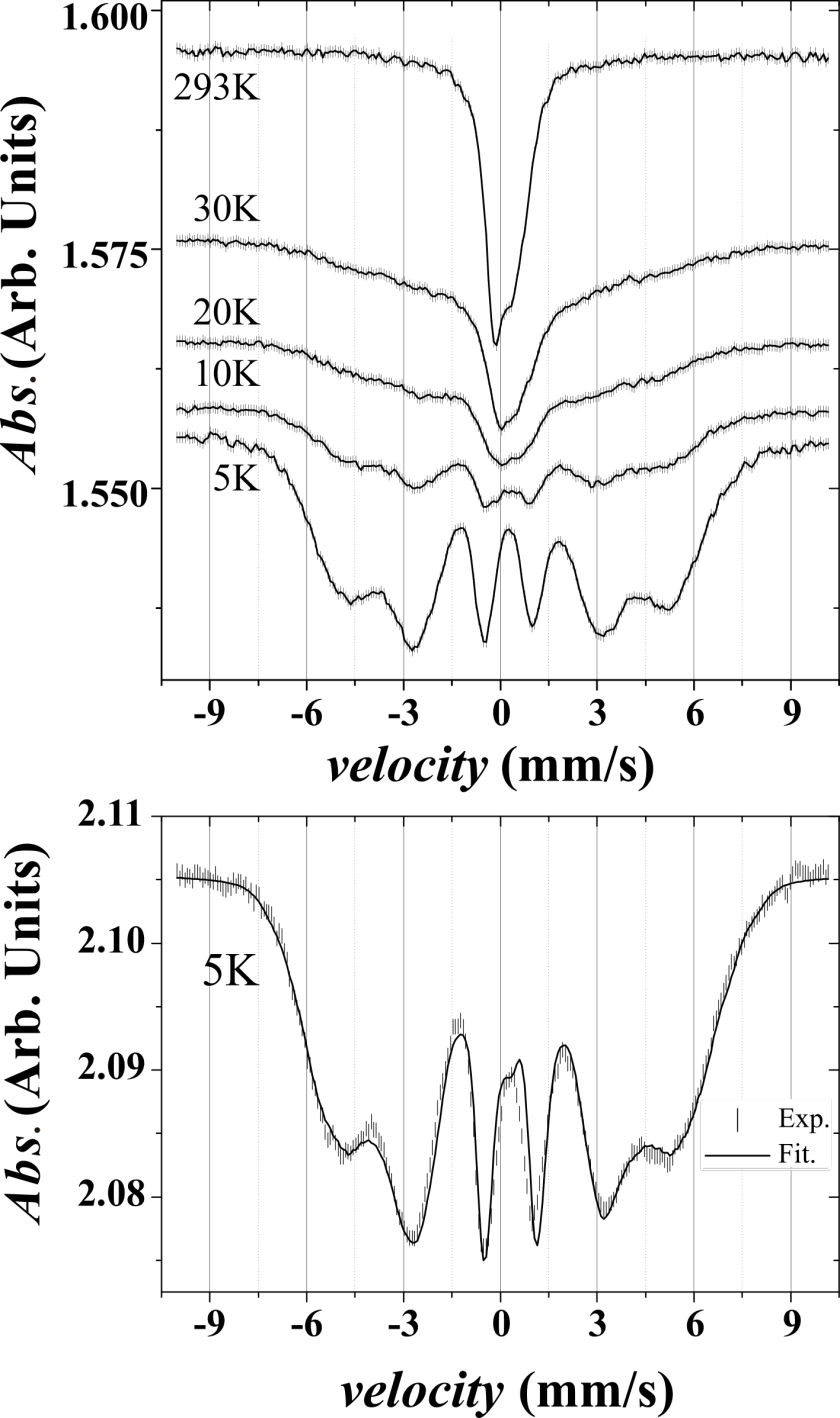
Top: Mössbauer spectra taken at different temperatures. Bottom: experimental spectra (symbols) taken at 5 K and the corresponding fit (solid line).

The Mössbauer spectra, recorded at various temperatures between 293 K and 5 K, are shown in Figure 4. The measurements were performed with a ^57^Co source in a Rh matrix and were calibrated against bulk α-Fe. Upon decreasing the temperature, the spectrum progressively splits but still remains broad, even at the lowest temperature. Such temperature dependence is characteristic of a superparamagnetic transition. The NPs, which have relaxation times (τ) longer than the measurement time (τ_m_), give rise to a sextet (blocked NPs). The superparamagnetic NPs with a short relaxation time (τ < τ_m_) show paramagnetic like behaviour. In the case of Mössbauer spectroscopy, τ_m_ is in the range of 10^−8^ s [33–35] and the superparamagnetic relaxation time is given by

[1]
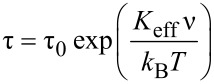


where ν is the volume, *K*_eff_ the effective anisotropy, and τ_0_ is of the order of 10^−11^–10^−9^ s [36]. The blocking temperature of the material corresponds to the temperature where the blocked and the superparamagnetic contributions are equivalent. We estimated it to be in the range of 25 ± 5 K.

**Figure 5:**
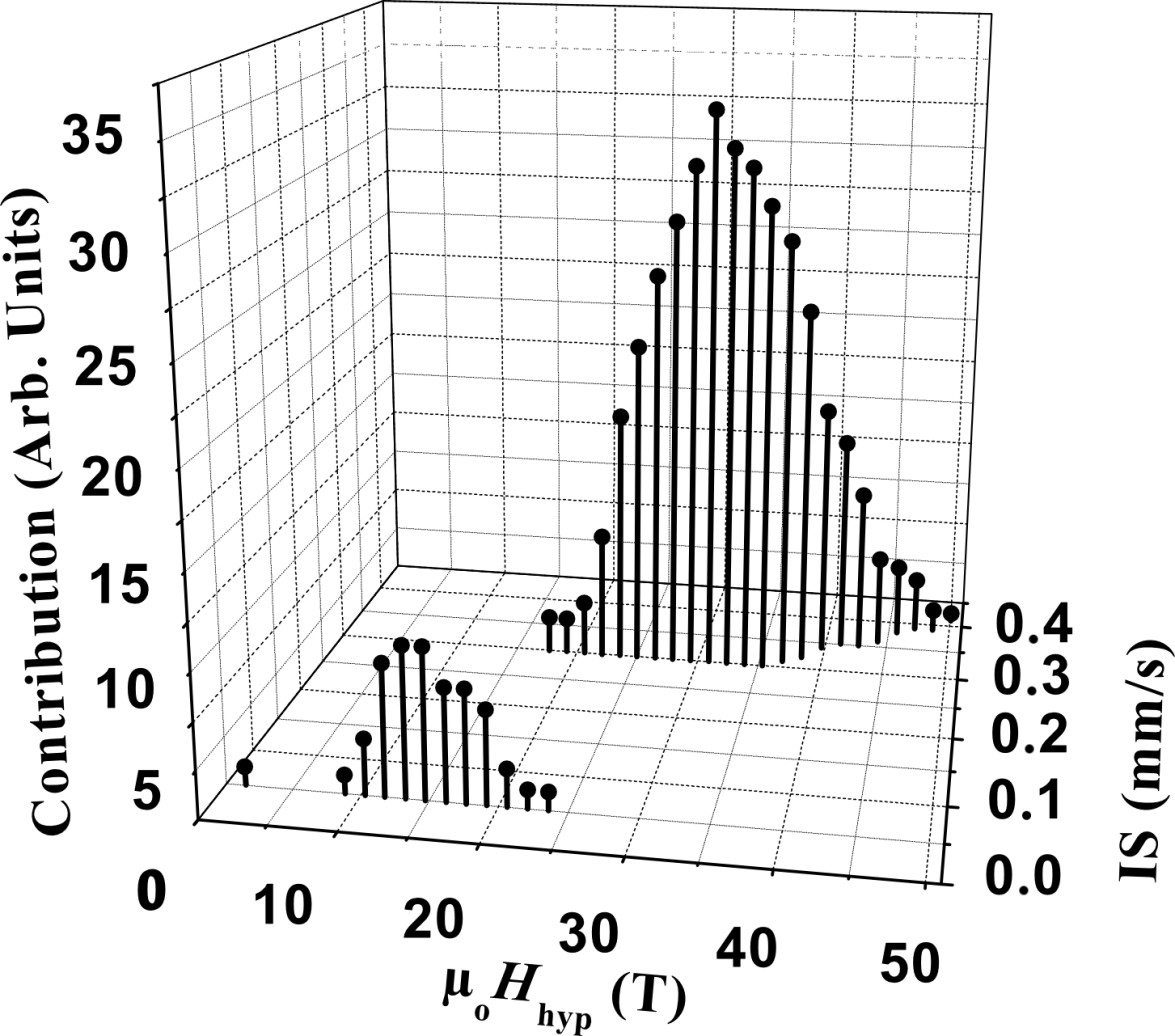
Distributions of the *IS* and the µ_0_*H*_hyp_ used to fit the experimental Mössbauer spectrum measured at 5 K.

We now focus on the analysis of the low temperature spectrum. At low temperature, relaxation phenomena on the time scale of Mössbauer spectroscopy should be negligible. The large broadening of the sextet is thus indicative of a distribution of hyperfine fields (*B*_hyp_). The fitting was carried out considering a distribution of hyperfine fields, with an isomer shift depending on *B*_hyp_ in order to adjust the experimental curve as precisely as possible. The relative areas of each component of the sextet have been constrained to the ratio of 3:2:1:1:2:3. Figure 5 displays the distributions of *B*_hyp_ and the corresponding isomer shifts (*IS*) used to simulate the spectrum measured at *T* = 5 K. Two distributions of *B*_hyp_ have been introduced. The first one, associated with a small *IS* centred on 0.05 mm/s, is composed of *B*_hyp_ values below 24 T. The second one has larger *B*_hyp_ values ranging from 20 T up to 50 T, associated with a larger *IS* centred around 0.35 mm/s. It is interesting to note that the *IS* increases with *B*_hyp_.

The parameters defining the main contribution are of the same order of magnitude as those measured on slightly larger metallic NPs. For α-Fe NPs with diameters in the range of 3.7 nm, Bødker and coworkers estimated the hyperfine fields to be in the range of up to 45 T with an average *IS* of 0.5 mm/s [11,37]. Furubayashi found a smaller *IS* of 0.22–0.27 mm/s for NPs with diameters of approx. 2.0 nm [10]. Mössbauer spectra measured on 1.8 nm metallic NPs stabilised by HN(SiMe_3_)_2_ display two similar Fe contributions, except that the upper limit of hyperfine field distribution is 42 T [25]. These effects – broad *B*_hyp_ distribution and large *IS* – may be related to the smaller size of our NPs and to the atomic polytetrahedral arrangement, in particular the presence of many non-equivalent Fe sites compared to the conventional α-Fe phase. Band structure calculations on cubic Fe phases show a shell dependent magnetic moment with quite large differences between the core and the surface [7–9]. The enhancement of the spin and orbital magnetic moments is progressive from the core to the surface as the coordination number decreases. On the surface, as a consequence of both the reduction of the coordination number and the interface with the vacuum, there is a reduction of the s-electron density and a larger local magnetic moment, leading to, respectively, an important increase of the *IS* combined with a larger hyperfine field [9–11,37–39]. This explains well our experimental results with a simultaneous increase of the isomer shift and hyperfine field. The polytetrahedral atomic arrangement should also play a role since it leads to a reduced coordination number and a large distribution in the Fe–Fe distances, even in the core as in the case of amorphous Fe which displays the same local structure [31]. This will lead to a very different electronic structure at each site, and to a much broadened dispersion of *B*_hyp_. Thus, we interpret the Mössbauer spectra as evidence of the progressive increase of the magnetic moment inside the particle from the core to the outer shell of the NP, the components corresponding to the large hyperfine fields with large isomer shifts being characteristic of surface atoms.

#### B. Magnetization

**Figure 6:**
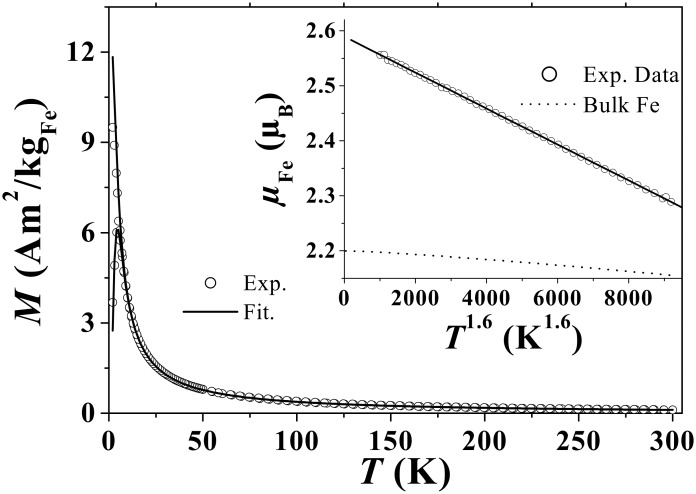
ZFC-FC magnetizations measured under µ_o_*H* = 1 mT. Inset shows the extracted temperature dependence of *M*_S_.

Magnetization measurements have been carried out with a commercial Quantum design SQUID magnetometer. Figure 6 shows the static zero-field-cooling field-cooling (ZFC-FC) magnetization curve versus temperature (*T*) in a low magnetic field of 1 mT. It exhibits a classical superparamagnetic (SP) transition with a blocking temperature *T*_B_ = 4.9 K.

**Figure 7:**
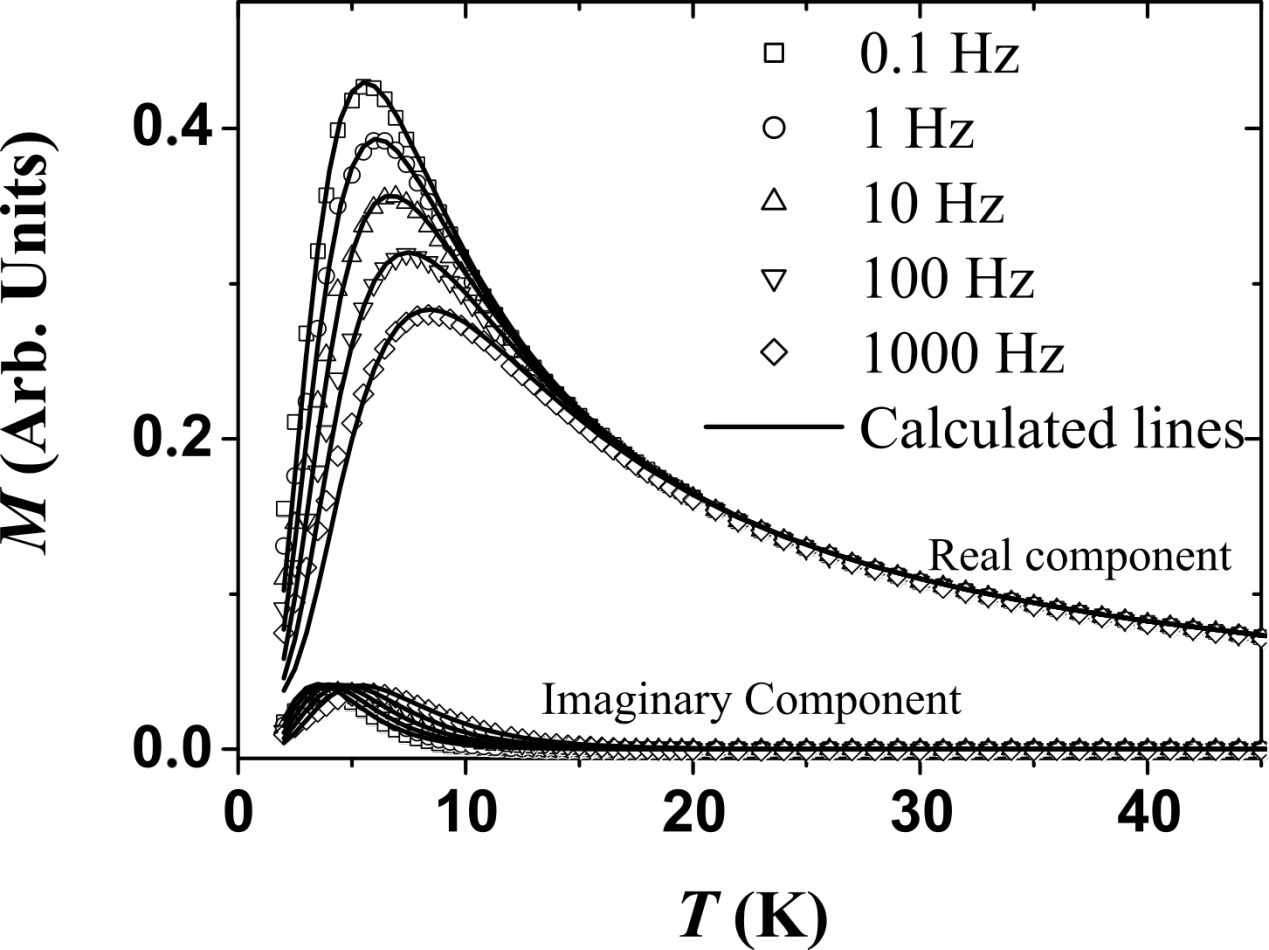
AC susceptibility measured for various frequencies (symbols) and their fits (solid lines).

The measure of the AC susceptibility (χ_AC_) shows the same superparamagnetic transition. Figure 7 displays the χ_AC_ variation versus temperature for a set of frequencies ranging from 0.1 Hz to 1000 Hz. The decrease of the measurement time τ_m_ induces an increase of *T*_B_.

**Figure 8:**
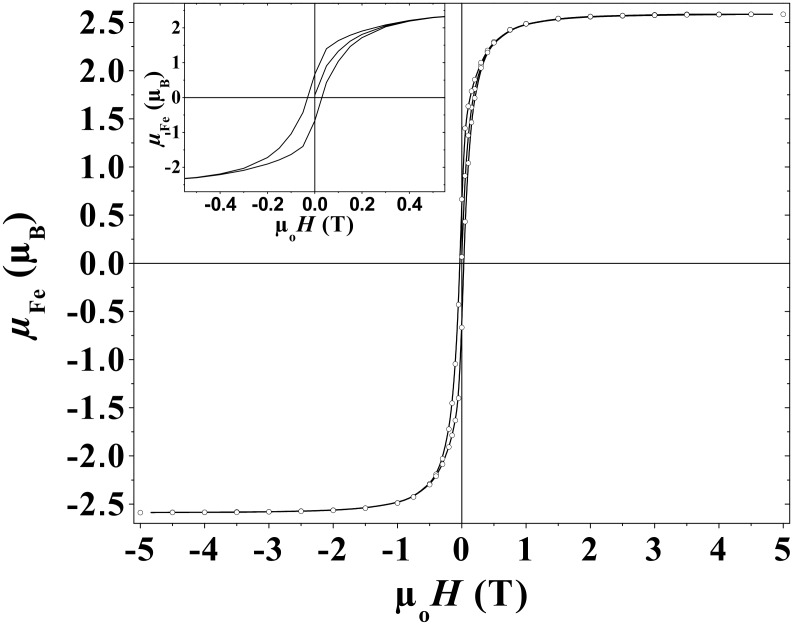
Hysteresis loop measured at 2 K. Inset: enlargement near zero field showing the coercive field.

**Figure 9:**
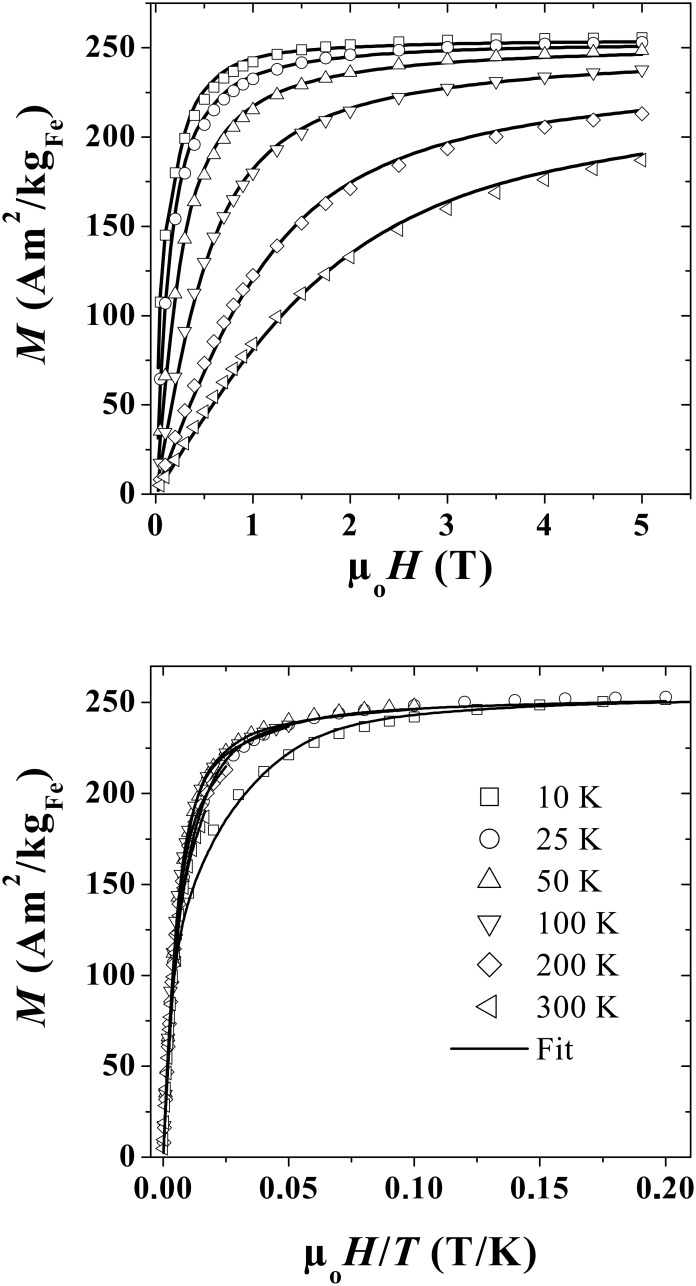
Magnetization curves in the superparamagnetic regime plotted versus the applied magnetic field (top) and versus the magnetic field divided by the temperature (bottom), for different temperatures.

Figure 8 and Figure 9 display the magnetization curves measured below *T*_B_ (*T* = 2 K) and above *T*_B_ (*T* = 10 K, 25 K, 50 K, 100 K, 200 K and 300 K), respectively. At *T* = 2 K (Figure 8), the magnetization is almost saturated in a field of 5 T, with a mean magnetic moment per Fe atom µ_Fe_ = 2.59 ± 0.05 µ_B_, well above the bulk value.

Above *T*_B_ (Figure 9, top), the hysteretic behaviour disappears, and the magnetization measured at 5 T progressively decreases with increasing temperature. The plot of these curves as a function of *H*/*T* exhibits some deviations from the pure Langevin behaviour (Figure 9, bottom). In low fields, the slope is practically the same for temperatures up to 100 K, and then starts to decrease as a consequence of the decrease of spontaneous magnetization (*M*_S_) with increasing temperature. Moreover, just below the magnetic saturation, some deviations arise, especially for the curve measured at *T* = 10 K (≈ 2 × *T*_B_), due to the influence of the anisotropy on the magnetization process [40].

The aim is now to determine a precise value of the effective anisotropy (*K*_eff_), and the evolution of the spontaneous magnetization (*M*_S_) with temperature. For an assembly of randomly oriented non-interacting particles in the superparamagnetic regime, the influence of the uniaxial anisotropy can be taken into account, leading to a modified Langevin function,

[2]
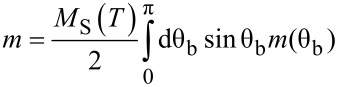


with

[3]
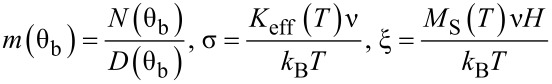


[4]
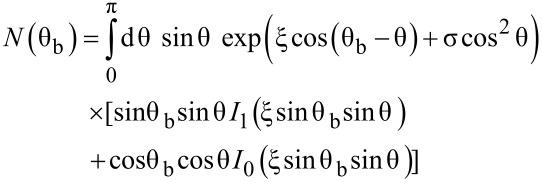


[5]
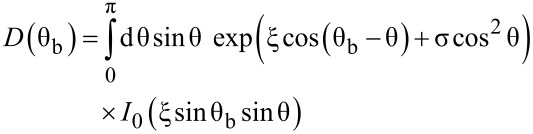


θ_b_ and θ define the angle of the applied magnetic field and magnetic moment with respect to the anisotropy axis [40], respectively. Interestingly, in low fields, for such an assembly of NPs, the susceptibility (χ = *m*/*H*) versus temperature follows a Curie law independent of the anisotropy [40]. It should be noted that this expression depends on *M*_S_ and *K*_eff_ which are both temperature dependent.

The static ZFC-FC curves can be modelled according to the usual expressions for non-interacting NPs with a uniaxial effective anisotropy including a log-normal size distribution [18],

[6]
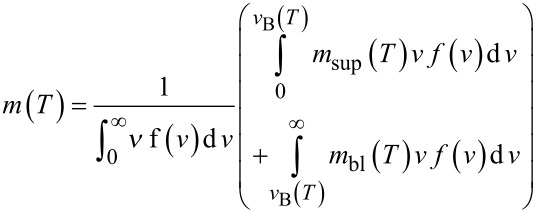


where


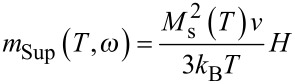


defines the superparamagnetic contribution, and where *m*_bl_ is defined as


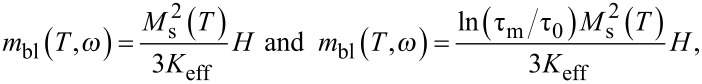


corresponding to the blocked contributions in the ZFC and FC experiments, respectively. 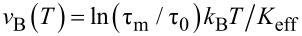 is the critical volume above (below) which the particles are in the blocked (superparamagnetic) state. This critical volume depends also on τ_o_, which is extracted from the dependence of *T*_B_ versus τ_m_ (see below). In the superparamagnetic state, the magnetization is given by,

[7]
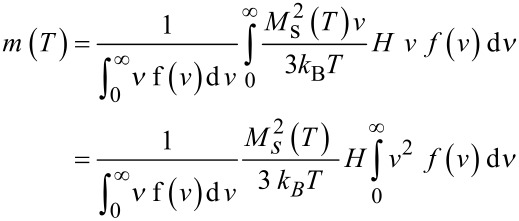


Thus, in the case of non-interacting NPs, the variation of the spontaneous magnetization versus the temperature can be extracted by plotting (*m*_Sup_Exp_* T*)^1/2^ versus *T*, where *m*_Sup_Exp_ corresponds to the ZFC-FC magnetization measured in the SP regime well above *T*_B_.

Finally, the ac-susceptibility can be modelled from the following expressions [41]:

[8]
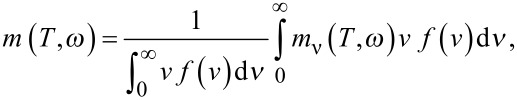


with

[9]
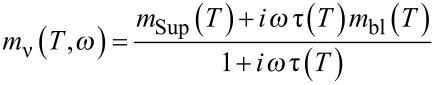


**Figure 10:**
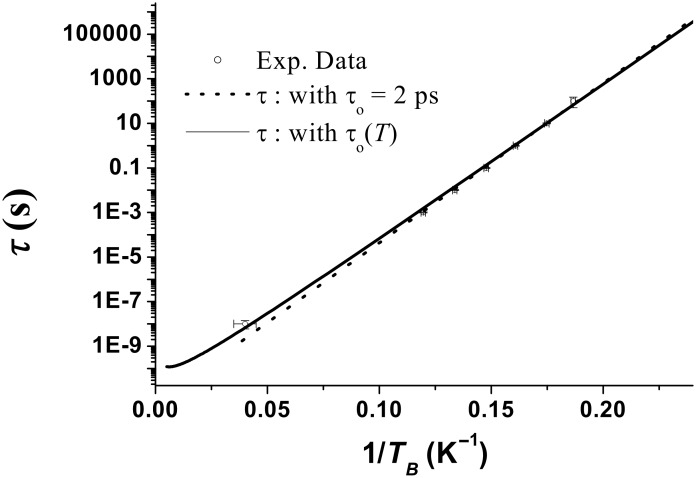
Relaxation time versus the inverse of temperature.

With these equations, it is possible to extract precisely the size distribution, the magnetic parameter *M*_S_(*T*) and the low temperature value of *K*_eff_. We first analyse the dependence of the relaxation time on temperature. Figure 10 displays the plot of log(τ_m_) versus 1/*T*_B_, this curve allows the determination of the pre-exponential time τ_o_ of the relaxation time τ. According to Equation 1, linear behaviour is expected. Fitting the variation of log(τ_m_) versus 1/*T*_B_ in the range of the longest measurement times gives τ_0_ = 2 ps. A deviation is observed for the shortest measuring times corresponding to the Mössbauer experiment. This deviation is reduced when the temperature dependence of τ_0_(*T*) 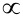 √*T* is taken into account [33–35,42]. This value is small compared to the expected, and usually measured, values, which are in the nanosecond range [43]. However, it is within the same range as Co NPs of similar size [44].

In a second step, we determine the temperature dependence of *M*_S_, from the static magnetization ZFC-FC curves by plotting *M*_S_(*T*) 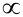 (*m*_Sup_Exp_* T*)^1/2^ versus *T*^n^.

As shown in the inset of Figure 6, *M*_S_ follows a *M*_S_(*T*) = M_S_(*T* = 0)[1−α*T*^n^] law, where n = 1.6 ± 0.05 and α = 3.27 10^−5^ K^−1.6^ allow the best fit. Even if the decrease of *M*_S_ is faster than in the case of bulk systems, as a result of lower surface coordination number [45], *M*_S_ remains above the bulk value, even at 300 K [46].

In a third step, knowing τ(*T*) and *M*_S_(*T*), the static ZFC-FC and the AC susceptibility curves (Figure 6 and Figure 7, respectively) are fitted with the same size distribution *f*(*v*) and *K*_eff_. As suggested by the narrow peak of the ZFC magnetization around *T*_B,_ the size distribution is very narrow (standard deviation of 0.15) centred on an average diameter of 1.6 nm. This magnetic size corresponds to that deduced from morphological studies by TEM. It approximately corresponds to clusters containing 150 to 200 atoms. Fitting these curves leads to an estimation of the effective anisotropy *K*_eff_ = (3.7 ± 1.0)·10^5^ J/m^3^ well above the bulk value. Another estimation of *K*_eff_ was obtained by fitting the magnetization curves in the superparamagnetic regime. In this case, the size distribution was not taken into account since its influence can be neglected. A slightly larger value, *K*_eff_ = (5.0 ± 1.0)·10^5^ J/m^3^, was obtained. Only the low temperature value of *K*_eff_ is accessible, since the influence of the anisotropy on the magnetization process rapidly vanishes and becomes negligible when *T* is above 10 × *T*_B_. Thus, we cannot access the temperature dependence of *K*_eff_.

In summary, the magnetization studies allow us to obtain some information on the magnetic size of these Fe NPs which contain 150–200 atoms on average. The mean magnetic moment per Fe atom µ_Fe_ = 2.59 ± 0.05 µ_B_, is much higher than the value for bulk iron (2.2 µ_B_), which well explains the strong hyperfine fields found with Mössbauer spectroscopy. The magnetic moment is higher than the one estimated by Furubayashi et al., who measured µ_Fe_ = 2.28 µ_B_ for particles with diameters around 2 nm [10], and the µ_Fe_ for Fe NPs stabilised by HN(SiMe_3_)_2_ [25]. It is, however, in good agreement with the values obtained for time-of-flight selected clusters by Billas et al. who measured µ_Fe_ in the range of 2.6–2.8 µ_B_ for clusters with less than 300 atoms (≤2 nm) [2]. This confirms that the synthesis using metal–organic precursors and an organic polymer as a matrix allows the growth of clusters with narrow size distributions and magnetic properties similar to those of free clusters, since the number of anchoring sites of the polymer on the surface is very small. The influence of surface coordination is thus limited. However, *M*_S_ decreases slowly with temperature in contrast to free-clusters [2]. It is difficult to interpret this latter effect, which is probably related to the structural order within the NPs. The origin of the enhancement of the magnetic moment must be related to the large surface to volume ratio. However, the adsorption during the synthesis of some hydrogen on the NP surface cannot be ruled out. Surface hydrides may form as has been demonstrated in the case of ruthenium [47]. This chemisorbed hydrogen could give a small spin contribution as evidenced with smaller clusters by Knickelbein and estimated to be 0.4 µ_B_ per adsorbed atom [48]. In our case, this contribution is not sufficient to explain the total magnetization per Fe atom observed, and the increase of the magnetic moment is thus related to an intrinsic effect as a consequence of the size reduction.

The value of the effective anisotropy *K*_eff_ in the range of (3.7–5.0)·10^5^ J/m^3^ is much higher than the bulk value, and larger than that deduced with micro-SQUID techniques from the magnetization curve of a single α-Fe NP containing 800 atoms embedded in a Nb matrix [49]. However, our result fits the diameter (Φ) dependence observed by Bødker et al., which follows *K*_eff_ = *K*_v_ + 6/Φ *K*_s_, with *K*_v_ = 3·10^4^ J/m^3^ and *K*_s_ = 0.09 mJ/m^2^ [12]. It is quite surprising that, whatever the surface state and the crystallographic order, a magnetic anisotropy of the same order of magnitude should be obtained (for comparison *K*_v_ ≈ 7·10^4^ J/m^3^ in bulk iron at low temperature). The origin of this enhancement is still an open question, since in the expression *K*_eff_ = *K*_v_ + 6/Φ *K*_s_, the second contribution has been derived considering a sphere, and a sphere should not induce any surface anisotropy. Most probably, with reduced size, deviations from sphericity become more important as a consequence of the presence of facets or incomplete surface layers, thus leading to a strong surface anisotropy.

#### C. FMR spectra

**Figure 11:**
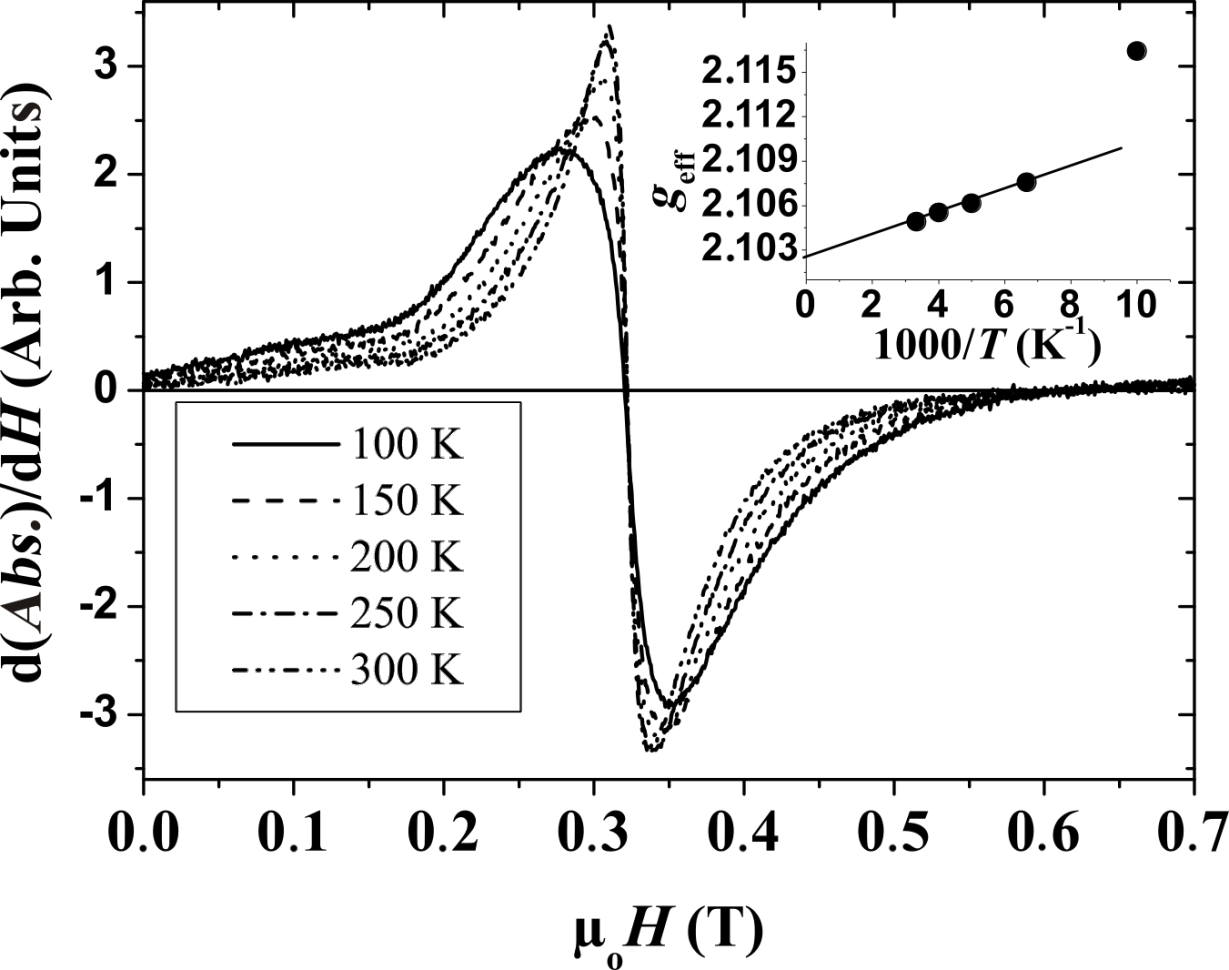
FMR spectra collected for various *T*. Inset displays the evolution of *g*_eff_ versus 1/*T*.

Ferromagnetic resonance experiments (FMR) have been performed in order to obtain some estimation of the relative contributions of the orbital and spin magnetic moments [50]. The most precise way to measure this ratio is to measure the frequency dependence of the resonant field [51–52]. We propose another approach based on the temperature dependence of the resonance field. Figure 11 displays the resonance curves, i.e., the derivative of the absorption line d(*Abs*.)/d*H,* measured at a frequency ω/2π = 9.5 GHz for several temperatures well above *T*_B_ in the SP regime. Upon decreasing the temperature, the absorption lines broaden, become inhomogeneous and shift toward low magnetic fields. The inset in Figure 11 displays the plot of the temperature dependence of the effective gyromagnetic ratio defined as 
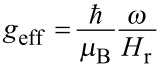
.

The resonant field (*H*_r_) was defined as d(*Abs.*)/d*H* = 0. At high temperature, *g*_eff_ displays a linear dependence when plotted as a function of *T*^−1^. Well above *T*_B_, the anisotropy field vanishes as a consequence of the SP behaviour. Under these conditions, we can demonstrate by expanding the relation defining the resonance condition [53], that *g*_eff_ = *g* (1 + A *T*^−1^), where A = *cte* × 2 *K*_eff_/5*k*_B_. *cte* is a coefficient depending on the orientation of the NP with respect to the applied field [54]. This extrapolation is only valid as long as ξ = *M*_S_*v*/*k*_B_*T* verifies the condition ξ < 1, which is not fulfilled when *T* is less than 100 K. Extrapolation towards *T*^−1^ = 0 K^−1^ leads to the gyromagnetic ratio *g* of the NPs. The value determined is 2.103 ± 0.001, close to the bulk value of 2.09. More precise measurements, especially at different frequencies, are required in order to confirm unambiguously if there is a small increase. Using this value, we estimate the ratio µ_L_/µ_S_ by the Kittel relation µ_L_/µ_S_ = (*g* − 2)/2 = 0.05 [55]. With these data, the values of average spin and orbital magnetic moments are estimated to be 2.46 µ_B_ and 0.13 µ_B_, respectively. This demonstrates that the enhancement of the total magnetic moment has contributions from both µ_L_ and µ_S_.

In comparison to the estimations made on other systems using XMCD [13–16], the average total magnetic moment per Fe atom is of the same order of magnitude. But, the ratio µ_L_/µ_S_ is smaller in our case. Band structure calculations are in relative good agreement with our estimations, for both the total magnetic moment and the ratio µ_L_/µ_S_. We believe that the small size of the particles compared to ours and the interactions with the substrates could lead to a stronger enhancement of µ_L_/µ_S_. In thin films, the magnetic anisotropy is related to the anisotropy of the orbital moment [56]. This anisotropy of µ_L_ cannot be measured in the case of disordered NPs randomly oriented. However, we believe that the large orbital contribution should be anisotropic, which could explain the large effective magnetic anisotropy measured in these particles. Oriented NPs would be necessary to investigate this latter phenomenon.

## Conclusion

Systems of well isolated metallic Fe NPs with diameters of less than 2 nm and embedded in a polymer have been synthesized by an organometallic approach. Structural studies reveal an unusual polytetrahedral atomic arrangement leading to locally both denser and less dense regions compared to the bulk phases. The large surface to volume ratio dominates the electronic properties and thus the magnetic properties. The total magnetic moment is increased since both the spin and orbital contributions are increased. Large hyperfine fields related to surface sites have been demonstrated, showing the influence of the reduction of surface coordination on the magnetic moment. Both the spin and orbital moments are also involved in this enhancement.
